# Mobile Eye Units in the United States and Canada: A Narrative Review of Structures, Services and Challenges

**DOI:** 10.3390/ijerph23010007

**Published:** 2025-12-19

**Authors:** Valeria Villabona-Martinez, Anna A. Zdunek, Jessica Y. Jiang, Paula A. Sepulveda-Beltran, Zeila A. Hobson, Evan L. Waxman

**Affiliations:** 1 Department of Ophthalmology, University of Pittsburgh School of Medicine, Pittsburgh, PA 15260, USA; villabonamartinev@upmc.edu (V.V.-M.); zdunek.anna@medstudent.pitt.edu (A.A.Z.); jiang.jessica@medstudent.pitt.edu (J.Y.J.); sepulvedabeltranp@upmc.edu (P.A.S.-B.); hobsonz@upmc.edu (Z.A.H.); 2 Department of Ophthalmology, University of Pittsburgh Medical Center, Pittsburgh, PA 15219, USA

**Keywords:** mobile eye care, mobile clinics, vision screenings, teleophthalmology, health disparities, outreach programs

## Abstract

**Highlights:**

**Public health relevance—How does this work relate to a public health issue?**

- Mobile Eye Units (MEUs) directly address persistent population-level barriers to eye care, transportation, insurance limitations, geographic distance, workforce shortages, and language or cultural challenges, that drive preventable vision loss in underserved areas of the U.S. and Canada.
- By bringing screenings, diagnostics, and referral pathways into community settings, MEUs serve groups with elevated risk of uncorrected refractive error, diabetic eye disease, glaucoma, and pediatric vision disorders, conditions that disproportionately affect marginalized communities.

**Public health significance—Why is this work significant to public health?**

- Vision impairment has broad public health consequences, affecting learning, employment, independence, and chronic disease management. Yet many communities lack accessible eye care infrastructure. MEUs offer a practical and scalable strategy to reduce these disparities.
- This review provides the first structured synthesis of MEU models in North America, describing their design, capabilities, limitations, and current gaps, and offering clear guidance on where services exist and where they are absent. By mapping MEU types, summarizing strengths and constraints, and outlining future directions, this work equips public health programs, health systems, and policymakers with a practical framework to identify needs and plan mobile eye care services.

**Public health implications—What are the key implications or messages for practitioners, policy makers and/or researchers in public health?**

- Practitioners and health systems can use the comparative model descriptions, tables, and geographic mapping in this review as decision-support tools to select the MEU type that best matches their community’s needs and operational capacity, and to strengthen integration of mobile eye care with navigation services and health-system referral pathways.
- Policymakers and researchers should prioritize standardized outcome reporting, improved follow-up systems, and evaluation frameworks to expand the evidence base for MEUs. Identifying gaps in geographic coverage and investing in interoperable digital systems, such as EHR-linked referrals, will be essential for strengthening continuity of care and ensuring mobile eye care programs are sustainable and scalable.

**Abstract:**

**Background and Objectives:** Mobile Eye Units (MEUs) have emerged as practical innovations to overcome geographic, financial, and systemic obstacles to eye care. Although numerous programs operate across the United States and Canada, a narrative review describing their structure, implementation and services, remain limited. This narrative review examines various MEUs models in the United States and Canada, using real-world examples to highlight each model’s structure, services, populations served, and key benefits and limitations. **Methods:** We performed a narrative review of peer-reviewed and gray literature published from 1990 to August 2025, identifying mobile eye units in the United States and Canada. Programs were grouped into four operational models based on services, equipment, and implementation characteristics. Ophthalmology residency program websites in the United States were also reviewed to assess academic involvement in mobile outreach. **Results:** We identified four operational MEU models: Fully Equipped Mobile Units (FEMUs), Semi-Mobile Outreach Units (SMOUs), School-Based Vision Mobile Units (SBVMUs), and Hybrid Teleophthalmology Units (HTOUs). FEMUs provide comprehensive on-site diagnostic capabilities but require substantial financial and logistical resources. SMOUs are lower-cost and flexible but offer more limited diagnostics. SBVMUs facilitate early detection in children and reduce school-based access barriers but depend on school coordination. HTOUs expand specialist interpretation through remote imaging, although their success relies on reliable digital infrastructure. Across all models, follow-up and continuity of care remain major implementation challenges. Approximately 21% of U.S. ophthalmology residency programs publicly report involvement in mobile outreach. **Conclusions:** MEUs play a critical role in reducing geographic and structural barriers to eye care for underserved populations across United States and Canada. However, limited outcome reporting, particularly regarding follow-up rates and continuity of care, hinders broader assessment of their effectiveness. Strengthening the integration of MEUs with patient navigators, integrated electronic health record, insurance support and support of local health networks is essential for improving long-term sustainability and impact.

## 1. Introduction

Vision is often overlooked in broader public health discussions, despite its influence on independence, education, employment, and safety. In North America, eye care systems face growing strain from workforce shortages [1,2], increasing eye diseases [3,4,5,6], limited specialist diversity [7,8], and well-documented disparities in access and outcomes [9,10,11,12,13,14,15]. These combined challenges continue to restrict timely access to eye care for underserved populations.

Mobile Eye Units (MEUs) have emerged as an adaptable strategy to reduce barriers to eye care. First introduced in Kenya and Canada in the 1950s [16,17,18], MEUs now operate worldwide, including operations in the United States, Europe, and Asia [19,20,21]. These units encompass a wide range of models, from vans and trucks to boats and aircrafts [22,23], and deliver services that span vision screenings, diagnostic imaging, and in some settings, procedures [24].

They have reduced disparities caused by geographic isolation, provider shortages, and financial or logistical barriers [25,26,27,28,29,30,31,32]. From fully equipped vans staffed by specialists to school-based programs and teleophthalmology-enabled outreach, these models have demonstrated increased screening rates, earlier detection of vision-threatening disease, and high patient satisfaction [21,32,33]. Despite their promise, comparative studies examining how different MEU models operate in North America remain limited [34,35,36,37,38,39,40,41,42,43,44,45,46,47,48,49,50,51,52,53,54,55,56]. Most available literature describes single programs, often affiliated with medical residency or nonprofit organizations, and provides little comparative analysis of service scope, population reach, or follow-up outcomes.

While many public health professionals recognize the barriers facing underserved communities, fewer may be familiar with how MEUs can effectively address these challenges in the context of eye care. This narrative review describes the range of MEU models currently operating across the United States and Canada. Drawing on real-world case examples, we aim to provide a practical framework to help academic departments, healthcare systems, and community organizations assess the feasibility and implementation of MEUs in their settings. As health systems continue to adapt to the realities of post-pandemic care delivery, mobile eye care represents a timely and promising avenue for advancing equity and prevention.

## 2. Materials and Methods

This study is a narrative review examining mobile eye units in the United States and Canada. A narrative approach was selected due to the heterogeneity of program designs, populations served, outcome measures, and reporting practices across the available literature.

We searched PubMed, Google Scholar, Web of Science, and publicly available institutional and agency reports for English-language sources published from January 1990 through August 2025. Core search terms included: “mobile eye unit,” “mobile eye care,” “mobile ophthalmology,” “mobile optometry,” “mobile vision screening,” “school-based eye clinic,” and “teleophthalmology” combined with “United States,” “Canada,” or “North America.” Reference lists of included articles were also screened for additional sources.

Sources were included if they (a) described a mobile or remote eye care delivery model and (b) provided sufficient operational detail to understand how the service was organized (setting, equipment, staffing, services, referral or follow-up processes). Peer-reviewed studies, program evaluations, and selected gray literature (e.g., program reports, government documents) were eligible. Commentaries without program details and technology papers lacking a care delivery model were excluded.

From each eligible source, we extracted descriptive information on model type, populations served, services offered, equipment and staffing, setting or partnerships, referral and follow-up pathways, reported outcomes when available, and any notes on funding or sustainability. Programs were then synthesized into four operational categories: Fully Equipped Mobile Units (FEMUs), Semi-Mobile Outreach Units (SMOUs), School-Based Vision Mobile Units (SBVMUs), and Hybrid Teleophthalmology Units (HTOUs). Given the variability in program structures and the absence of standardized outcome reporting, no formal quality appraisal, statistical comparison, or meta-analysis was performed. Instead, we opted for a descriptive presentation of the information rather than performing statistical analyses.

To assess the presence of mobile outreach within academic settings, we reviewed publicly available websites of U.S. ophthalmology residency programs to identify references to mobile units, teleophthalmology vans, or outreach clinics as part of training or community service.

As a narrative review drawing partly on gray literature, this work is not exhaustive. Program descriptions, reporting practices, and operational models vary in depth and may not reflect current activities.

## 3. Results

Mobile Eye Care Units (MEUs) are organized programs or services that deliver essential eye health care directly to individuals in underserved or hard-to-reach communities, operating outside traditional clinic settings. These units may comprise fully equipped vehicles, portable outreach setups, or telehealth-enabled platforms, and are staffed by professional eye care teams to provide screenings, diagnostic services, education, and referrals.

Before describing each model, it is important to note that each MEU model aims to address persistent gaps in eye care access driven by geographic distance, lack of insurance, workforce shortages, language and cultural differences, and other structural barriers commonly identified in the literature [10,11,12,13,14,15,25,26,27,28]. By providing services in community-based settings, MEUs expand access to screenings, diagnostics, referrals, health education and play a critical role in overcoming barriers to eye care.

### 3.1. Types of MEU Models

**Figure 1:**
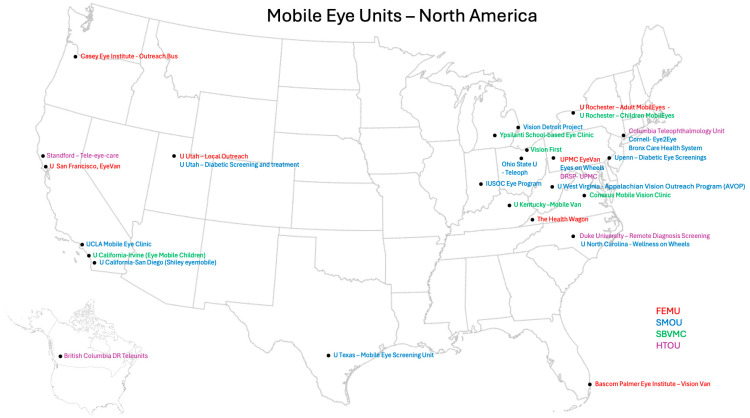
Geographic map of Mobile Eye Units (MEUs) identified in the United States and Canada, categorized by operational model and location. Fully Equipped Mobile Units (FEMUs) are shown in red, Semi-Mobile Outreach Units (SMOUs) in blue, School-Based Vision Mobile Units (SBVMUs) in green, and Hybrid Teleophthalmology Units (HTOUs) in purple. The figure includes both ophthalmology residency–affiliated and independent programs, as identified through the literature and gray sources. This visualization illustrates the distribution and diversity of MEU models across North America.

**Figure 2:**
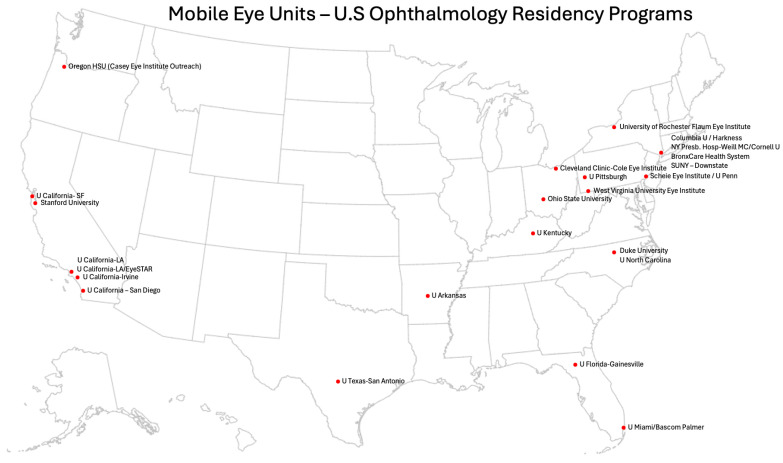
Ophthalmology residency programs in the United States that report an affiliated Mobile Eye Unit (MEU) or structured mobile outreach effort. Of 119 residency programs reviewed, 25 (21%) listed a mobile outreach component on their websites. Programs are displayed by state, highlighting limited but growing integration of mobile eye care into residency training and departmental outreach.

Our review identified 31 Mobile Eye Unit (MEU) programs operating across the United States and Canada. Among U.S. academic centers, 25 of 120 ophthalmology residency programs (21%) reported involvement with a mobile clinic or outreach initiative. Please refer to Figure 1 for a geographic map of all MEUs identified in this review across the U.S. and Canada, organized by model type (FEMU, SMOU, SBVMC, HTOU). Residency-affiliated programs are further detailed in Figure 2, which summarizes U.S. ophthalmology residency programs with mobile outreach components. The complete list of programs, including state-by-state information and website links, is provided in Appendix A. An additional six non–residency-affiliated programs were identified through gray literature and institutional sources.

Across these programs, four recurring MEU models were observed:

1. **Fully Equipped Mobile Units (FEMUs)**
2. **Semi-Mobile Outreach Units (SMOUs)**
3. **School-Based Vision Mobile Units (SBVMUs)**
4. **Hybrid Teleophthalmology Units (HTOUs)**

These categories reflect patterns described in the literature and represent common structural and operational configurations of MEUs in North America. Please refer to Table 1 for a comparative summary of the four MEU models across key operational dimensions. In addition, Appendix B provides detailed case examples outlining services, equipment, follow-up mechanisms, and implementation challenges for representative programs within each model.

#### 3.1.1. Fully Equipped Mobile Health Units (FEMU)

- **Description**: Self-contained eye clinics built into large vehicles: buses, RVs, or trailers. These units typically have power supply, climate control, and internet connectivity. They are often affiliated with academic centers, public health departments, or non-profits. Typical staffing includes ophthalmologists or optometrists, technicians, trainees, and support staff. Unlike Semi-Mobile Outreach Units (SMOUs), FEMUs deliver care inside the vehicle itself, where diagnostic equipment is permanently installed and not transported to host sites.
- **Services Provided**: Visual acuity testing, refraction, intraocular pressure checks, slit-lamp examination, fundus photography, and, in some models, optical coherence tomography (OCT). Some FEMUs provide dispensing of glasses, medications, and referrals for subspecialty or surgical care.
- **Settings and Communities Served**: Rural towns, low-income urban neighborhoods, Indigenous communities, senior living facilities, and areas without fixed eye care infrastructure.
- **Strengths**: High diagnostic capability in a single setting, earlier detection of chronic disease, reduced transportation burden, and ability to serve high-risk populations.
- **Limitations**: High costs for vehicles, equipment, staffing, fuel, and maintenance. Parking and permitting can be challenging, weather can disrupt operations, and units are not suitable for intraocular surgery.

##### Representative Case Examples

- **Casey Eye Institute Outreach Program [35,36]**

Established in 2010, this FEMU operates a fully equipped outreach bus containing two exam lanes with slit lamps, autorefractors, tonometers, and integrated EHR. Staffed by ophthalmologists, residents, and volunteers, the program delivers no-cost care in partnership with public health organizations. It effectively reduces geographic barriers for rural communities but faces sustainability challenges due to episodic delivery, operational costs, and reliance on philanthropic support.

- **University of Pittsburgh Medical Center (UPMC) eyeVan**

Launched in 2023 by the UPMC Vision Institute, the eyeVan is a fully equipped mobile clinic that combines features of both FEMU and SMOU models. The unit includes a phoropter, slit lamp, indirect ophthalmoscope, fundus camera, OCT machine, and mobile power and internet. Staffed by ophthalmologists, optometrists, technicians, and trainees, it provides visual acuity testing, intraocular pressure checks, dilated exams, imaging, and eyeglass prescriptions through an optical partner.

The eyeVan travels to schools, senior facilities, and community health centers across Western Pennsylvania and can also be deployed in SMOU mode by setting up portable stations inside buildings. Patient navigators help coordinate follow-up and financial assistance. While effective in reaching mobility-limited or underserved populations, the program depends on philanthropic support and faces logistical challenges such as equipment maintenance and limited operating days.

- **The Health Wagon [37]**

Founded in the 1980s in Appalachian Virginia, this hybrid FEMU–SMOU model uses RV-based medical units to deliver primary and specialty care, including periodic vision services, in remote rural communities. Outreach events are typically held in schools, community gyms, or temporary clinic sites. Services include refraction, vision screenings, and diabetic retinopathy evaluations. The program improves access for geographically isolated patients but relies heavily on volunteers and experiences challenges with continuity of follow-up care.

#### 3.1.2. Semi-Mobile Outreach Programs (SMOU)

- **Description**: Mobile outreach teams bring portable ophthalmic equipment to temporary clinic locations such as community centers, shelters, churches, FQHCs, and health fairs. Care is delivered inside the host site, not in a dedicated vehicle, and equipment is set up and packed down at each event.
  Unlike Fully Equipped Mobile Units (FEMUs), SMOUs do not contain built-in clinical space or permanently mounted diagnostic instruments; all equipment is transported and assembled at the outreach location.
- **Services Provided**: Vision screenings, auto/manual refraction, trial lens fitting, intraocular pressure measurement, screening for cataracts, glaucoma, and diabetic retinopathy, patient education, and written referrals for follow-up care.
- **Settings and Communities Served**: Urban and peri-urban communities, including individuals experiencing homelessness, migrant workers, and uninsured or underinsured populations.
- **Strengths**: Flexible and lower cost compared to FEMUs; can operate without on-site plumbing or electricity; easily deployable in a variety of settings; culturally adaptable; and supports community-based training for students and trainees.
- **Limitations**: Portable equipment limits diagnostic capacity; coordination and follow-up often occur off-site; documentation may be paper-based or fragmented; and operations frequently rely on volunteers and local partnerships.

##### Case Examples

- **University of California Los Angeles (UCLA) Mobile Eye Clinic [38,39]**

Established in 1975 by the UCLA Stein Eye Institute, this program operates entirely with portable ophthalmic tools transported to schools, shelters, and community centers. It provides screenings, refractions, and eyeglass distribution across underserved areas of Southern California. The clinic has served more than 300,000 individuals and depends on philanthropic partnerships for sustainability.

- **Eyes on Wheels [40]**

Founded in 2005 (formerly the Guerrilla Eye Service), this UPMC-affiliated initiative deploys ophthalmologists, residents, and medical students with portable equipment to FQHCs, shelters, churches, and community centers. Clinics occur during evenings and weekends and offer dilated exams, refractions, tonometry, glasses, and referrals. Care is provided free of charge. While the program improves continuity by linking patients to UPMC services, it depends on volunteers, equipment maintenance, and temporary infrastructures.

- **Indiana University Student Outreach Clinic Eye Program (IUSOC) [41]**

Established in 2009, this student-run clinic operates periodically at a fixed site using portable tools. Services include visual acuity testing, intraocular pressure measurement, refraction, and referrals. Optometry and medical students provide care under ophthalmologist supervision, serving primarily uninsured residents in Indianapolis.

- **Kresge Eye Institute (KEI) Vision Detroit Project [42]**

Launched in 2016, this SMOU model partners with community organizations to host pop-up clinics across Detroit. It focuses on underserved and minority populations by offering exams, glaucoma and diabetic retinopathy screenings, and eyeglass prescriptions. Strong partnerships with community groups support navigation and follow-up efforts.

#### 3.1.3. School-Based Vision Mobile Units (SBVMU)

- **Description**: School-Based Vision Mobile Units (SBVMUs) deliver eye care directly within school environments, either by bringing a mobile van onto school grounds or by setting up temporary exam stations inside school buildings. These programs focus primarily on children who fail school vision screenings and need full exams and eyeglasses. Unlike FEMUs, which operate as comprehensive mobile clinics for the general population, and unlike SMOUs, which serve varied community sites, SBVMUs are purpose-built around school workflows and pediatric needs, with services scheduled during the academic day.
- **Services Provided**: Repeat vision testing, cycloplegic refractions, on-site dispensing of prescription glasses, family education, and referrals to pediatric ophthalmology or optometry when needed.
- **Settings and Communities Served**: Elementary and middle schools in low-income districts, rural areas, or regions with historically low follow-up after school screenings.
- **Strengths**: Eliminate transportation barriers and the need for parental time off; support academic performance by addressing undiagnosed vision-related learning issues; and foster continuity by engaging school nurses, counselors, and support staff.
- **Limitations**: Require coordination with school administration; cannot serve students absent on clinic days; limited to non-complex conditions; and sustainability often depends on local funding or grants.

##### Case Examples

- **Vision First [21,43]**

Established in 2002 by the Cole Eye Institute, this RV-based program visits more than 80 schools each year in the Cleveland Metropolitan School District. It provides two-stage exams with cycloplegic refraction and delivers custom eyeglasses directly to schools. The program has identified significant vision problems in thousands of underserved students and operates through a partnership-based funding model.

- **Conexus Mobile Vision Clinic [44]**

A nonprofit program offering full-service mobile eye clinics across Virginia. Staffed by optometrists and technicians, Conexus provides comprehensive exams, eyeglass prescriptions, and glasses delivery within two weeks. It improves access in rural and low-income school districts, though long-term follow-up remains a challenge.

- **Ypsilanti School-Based Eye Clinic [45]**

A collaboration between the University of Michigan and Ypsilanti Community Schools, this semi-mobile program offers in-school exams, glasses, and care coordination for high-risk students. It improves early detection and academic performance but is limited by school scheduling constraints and funding variability.

- **Additional case examples are**: The Philadelphia Eagle Eye Mobile which provides optometric vision care to children who fail a vision screening performed by nurses at schools in low-income areas [46]. UCLA mobile provides vision screenings at public neighborhood elementary schools and community centers [39]. A nonprofit organization in Virginia provided in-school instrument-based screening and noncycloplegic examinations and refractions in elementary, middle, and high schools [47]

#### 3.1.4. Hybrid Teleophthalmology Models (HTOU)

- **Description**: Hybrid Teleophthalmology Units (HTOUs) combine on-site imaging and basic screening with off-site diagnostic interpretation by eye specialists. Technicians or community health workers collect fundus photos or OCT images in the field, but diagnosis, grading, and management recommendations come from remote ophthalmologists or optometrists.
  Unlike SMOUs, HTOUs do not perform full clinical exams on-site, and they rely heavily on remote specialist input for clinical decision-making and follow-up plans. Unlike FEMUs, HTOUs do not operate a fully equipped vehicle; instead, they use portable devices paired with telemedicine platforms.
- **Services Provided**: High-resolution fundus photography; OCT where available; intraocular pressure assessment; asynchronous or synchronous specialist review; patient counseling; and follow-up navigation. Some programs incorporate digital tools to streamline triage and referral.
- **Settings and Communities Served**: Remote Indigenous communities, geographically isolated health centers, primary care clinics, and urban neighborhoods with limited in-person specialist access.
- **Strengths**: Extends specialist capacity across distances; supports early detection and triage with minimal on-site resources; scalable with lower long-term cost per patient; and aligns well with public health initiatives.
- **Limitations**: Requires reliable broadband connectivity and digital infrastructure; technicians need training for imaging and software use; asynchronous review may introduce delays; and start-up costs may be prohibitive for smaller organizations.

##### Case Examples

- **Columbia University Teleophthalmology Unit [48]**

Launched in 2019, this program deploys portable diagnostic equipment to public housing sites and community centers. Technicians capture images that are reviewed remotely within 48–72 h by ophthalmologists. Patients receive follow-up calls and are connected to appropriate care. The model supports early disease detection and builds community trust but depends heavily on connectivity and coordinated follow-up.

- **British Columbia’s Diabetic Retinopathy Teleunits [49]**

Established in the early 2000s, these units serve remote and Indigenous communities by deploying technicians to conduct fundus photography and upload images for specialist review in urban centers. The program improves capacity for diabetic eye care and reduces preventable vision loss but is limited by infrastructure gaps and challenges in sustaining patient engagement.

- **Diabetic Retinopathy Screening Program—University of Pittsburgh Medical Center [50]**

Established in 2008, this system placed approximately 40 fundus cameras across UPMC primary care and community sites. Local personnel were trained to capture retinal images, which were uploaded for asynchronous interpretation by UPMC specialists. The program expanded screening access and built workforce capacity but faced challenges including staff turnover, equipment maintenance, and barriers to follow-up.

## 4. Discussion

Mobile Eye Units (MEUs) offer diverse operational advantages that make them effective in addressing access barriers across different populations and geographic settings in the United States and Canada. Although their structures vary, MEUs share a common goal: delivering essential vision services directly to communities that face significant barriers to traditional eye care.

### 4.1. Four Primary MEU Models Identified

This review identified four main MEU models currently operating in North America: Fully Equipped Mobile Units (FEMUs), Semi-Mobile Outreach Units (SMOUs), School-Based Vision Mobile Units (SBVMUs), and Hybrid Teleophthalmology Units (HTOUs). Each model presents distinct strengths and trade-offs.

FEMUs closely simulate fixed-site clinical environments and deliver comprehensive diagnostic capacity on-site, making them ideal for rural or hard-to-reach regions. SMOUs provide greater flexibility and lower operational costs but rely heavily on partnerships with host sites and may have limited continuity of care. SBVMUs focus on school-aged children, helping reduce vision-related learning barriers by delivering exams and glasses within school settings, though these programs often require extensive coordination and sustainable funding. HTOUs expand access to subspecialists through asynchronous or synchronous image review but depend on reliable broadband connectivity, trained staff, and robust follow-up pathways.

### 4.2. Importance of Model Selection Adapted to Community Needs

Model selection should be guided by population needs, available staffing and infrastructure, and the desired scope of services.

- FEMUs are most appropriate when comprehensive exams and diagnostics are needed on-site.
- SMOUs are ideal for rapid deployment in partner settings such as clinics or shelters.
- SBVMUs are effective for providing care to children within school systems.
- HTOUs are best suited for situations requiring remote subspecialty input, where portable imaging can be paired with structured pathways for transmitting results and coordinating follow-up care.

### 4.3. Major Problems in MEU Programs Requiring Attention

Despite their differences, all MEU models share several key benefits: expanded geographic reach, culturally competent service delivery, and increased screening and referral rates. However, persistent cross-cutting challenges remain. These include gaps in follow-up tracking, limited outcome reporting, funding problems, staffing, and lack of integration with electronic health records, health systems, or insurance processes, all of which undermine continuity of care and long-term impact.

Unfortunately, despite all efforts in initial screening provided by these mobile units, insufficient follow-up programs remain a challenge. As an example, few peer-reviewed studies describe MEU outcomes or implementation frameworks in detail. As noted by Fu et al. [33] and confirmed in our review, outcome reporting is inconsistent and often absent. In our search of ophthalmology residency programs in the U.S, only 25/119 programs (21%) listed a mobile outreach component on their websites, and even fewer shared data on program performance 7/25 (28%) [21,35,38,39,40,41,42,50]. Strengthening referral handoffs, documenting outcomes, and improving interoperability in the healthcare system are essential next steps for program development and evaluation, particularly for underserved and geographically isolated populations.

### 4.4. Advice for Improvement of MEUs

Improved outcome tracking, including follow-up rates, disease detection, and patient satisfaction, should support the replication of successful models. Platforms such as the American Telemedicine Association’s Ocular Community [51] and the Mobile Health Map [52] could serve as repositories for shared data and protocols. Currently, most MEU outcomes and implementation strategies are still not publicly reported. Although none of the MEU programs identified in this review reported the integration of artificial intelligence (AI), future integration of AI-assisted triage or image analysis may enhance efficiency as digital infrastructure evolves.

Now, the most robust outcome data are available from SBVMUs. For instance, the Eagles Eye Mobile in Pennsylvania reported a 53% follow-up rate [46]. Broader adoption of such reporting practices would significantly enhance the field. The importance of early detection and follow-up is further highlighted by findings from Traboulsi et al. [43], who reported that approximately 10% of 5- and 6-year-olds screened in school settings required glasses or treatment for amblyopia or strabismus. These prevalence rates remained stable over 12 years [21,43], demonstrating the importance of sustained pediatric screening programs. However, the authors did not provide data on referral outcomes following the initial screenings or on the specific pathologies identified.

Unfortunately, despite all efforts in initial screening provided by these mobile units, insufficient follow-up programs remain a challenge. As an example, The Baltimore Vision Screening Project [53,54,55] reported low follow-up rates even when services were provided at no cost. Effective school-based programs must coordinate closely with school calendars and staffing and incorporate strategies to address these barriers. Future implementation research should explore these gaps.

MEU initiatives should not remain isolated, temporary efforts but should instead be embedded within larger healthcare systems to ensure long-term sustainability and scalability. Barriers to eye care exist across North America, regardless of geography, insurance status, or health literacy, highlighting the need for institutional commitment. Developing regional MEU networks supported by interoperable data platforms, public health agencies, community health workers, and academic partnerships could transform mobile outreach into a permanent, equitable component of the eye care infrastructure.

### 4.5. Strategies to Improve Follow-Up, Care Continuity, and Future Directions for MEUs

To improve follow-up rates after a pathology is identified, Dotan G. et al. demonstrated that integrating social worker support increased follow-up completion from 5% to 59%. They also reported that patients who returned for care were significantly more likely to be insured (73%) than uninsured (14%), underscoring insurance status as a major barrier to continuity of care [56]. Public health and organizational efforts should therefore prioritize expanding access to insurance and strengthening systems that help patients navigate coverage requirements.

Health literacy regarding insurance and vision benefits also remains a critical challenge. In a Philadelphia survey, many caregivers were unfamiliar with the ophthalmic services available under Medicaid, contributing to delays or refusal of follow-up care [57]. This highlights health literacy as a modifiable barrier that can be addressed through targeted education and clearer communication.

Other well-documented barriers, including language differences, transportation limitations, low general health literacy, and restricted availability during clinic hours [9,10,11,12,13,14,15,25,26,27,28,29,30,31,32,58,59,60], can be mitigated through strategies such as professional interpretation services, deployment of MEUs in community settings, engagement of community health workers, and participation in established outreach events such as Give Kids Sight Day [56] and Mission of Mercy [10]. Because many of these programs operate on weekends, they improve accessibility for working families and increase the likelihood of completing follow-up care.

Looking ahead, we propose that MEU initiatives should be integrated within larger healthcare systems rather than operating in isolation. Such integration would enhance long-term follow-up care coordination, strengthen sustainability, and support scalability. Strategies such as adopting interoperable referral pathways, enhancing data exchange capabilities, or incorporating partial EHR connectivity could support more efficient tracking and reduce attrition. Given the substantial variability in outcome reporting across programs, future research should also aim to develop a standardized core outcome set for MEUs, including measures such as disease-detection yield, follow-up adherence, patient-reported outcomes, and cost-effectiveness, to guide evaluation and system-level improvement. Although none of the programs included in this review reported the integration of artificial intelligence (AI), AI-assisted triage or automated image analysis may represent a future opportunity to enhance efficiency and reach, particularly within teleophthalmology and school-based screening models, as digital infrastructure continues to evolve.

### 4.6. Considerations for Mobile Eye Surgical Units (MESUs)

A notable absence in our North American review was Mobile Eye Surgical Units (MESUs) or “eye camps,” which are widely used in countries like India for cataract and other high-volume procedures. These mobile surgery programs, used by both domestic teams and groups such as the U.S. Air Force in Central and South America, have demonstrated high surgical output in resource-limited settings outside the U.S. [61,62,63,64]. Their absence in the U.S. likely reflects a combination of regulatory, reimbursement, and infrastructure barriers.

International findings suggest potential value. For example, a 2023 study in Malaysia found that implementing a mobile cataract program improved the country’s Effective Cataract Surgical Coverage (eCSC), a metric endorsed by the WHO. While eCSC indicators exist for Latin America, Asia, and Africa [65,66,67], similar data are lacking in the U.S. Developing national eCSC benchmarks could support efforts to assess and improve surgical equity domestically.

Evaluating the cost-effectiveness and safety of mobile surgical models in high-income countries is equally important. While India’s MESUs have long demonstrated success [68], similar initiatives in Switzerland have proven safe and efficient despite a vastly different healthcare system [69]. These findings highlight the need for context-specific implementation studies to explore how such models might be adapted to the U.S. environment. That said, U.S. ASC conditions, state licensure requirements, certificate-of-need laws, and building code constraints currently make mobile surgical units impractical in most regions.

## 5. Limitations

As a narrative review, this study has inherent limitations. The available literature on MEUs in North America is heterogeneous, with significant variation in program structure, reporting practices, and outcome measures. Many programs lack publicly available or standardized data, particularly regarding follow-up outcomes, referral completion, and long-term sustainability. Because of these inconsistencies, we did not conduct a formal quality appraisal or meta-analysis; instead, findings were synthesized descriptively to highlight model characteristics and implementation considerations. In addition, some information relied on gray literature and institutional reports, which may be incomplete or outdated. These limitations underscore the need for improved reporting and rigorous evaluation of MEU programs.

## 6. Conclusions

This review demonstrates that mobile and tele-enabled models can deliver eye care effectively within the communities where patients live, learn, and receive other services. The choice of MEU model should be guided by local needs, available staffing, resources, and the capacity to ensure continuity of care after the initial visit. A practical first step is to launch a small pilot, track a few meaningful outcomes, such as glasses delivered, referrals completed, and return of results, and adjust workflows with host partners. Sharing concise, comparable reports and step-by-step procedures can help other sites replicate successful strategies. When embedded within a health system and supported by interoperable digital infrastructure, MEUs can expand equitable eye care delivery across the United States and Canada without requiring new fixed facilities.

## Figures and Tables

**Table 1:** Summary Comparison of Mobile Eye Unit (MEU) Models and Their Operational Characteristics. This table provides a synthesized comparison of the four primary MEU models described in the manuscript. It consolidates shared and distinguishing features across models: vehicle configuration, the level of equipment integration, typical staffing, diagnostic capabilities, portability, and ideal deployment settings. Cost estimates and scalability metrics are intentionally excluded due to inconsistent or absent reporting across programs. This descriptive comparison is intended to support decision-makers, academic centers, and community organizations in identifying the model best suited to their operational context. FEMU: Full-Equipped Mobile Units, SMOU: Semi-Mobile Outreach Units, SBVMC: School-Based Vision Mobile Units, HTOU: Hybrid Tele-Ophthalmology Units.

Dimensions	FEMU	SMOU	SBVMU	HTOU
Vehicle/Structure	Large dedicated vehicle with built-in-clinic	Set-up inside the clinic; portable equipment packed in the van	Van or school-site temporary clinic	Portable imaging + telemedicine platform
Staffing	Ophthalmologist/Optometrist + techs + trainees	Ophthalmologist/Optometrist + techs + trainees	Pediatric ophthalmologist/optometrist + tech + school nurses/techs	Tech for imaging + remote ophthalmologist/optometrist
Diagnostic Capability	High (slit lamp, fundus camera, OCT)	Moderate (portable tools)	Moderate-high (pediatric focused equipment)	High for imaging; dependent on remote interpretation
Portability	Low	High	Moderate	High
Ideal Setting	Rural, remote or medically underserved areas needing comprehensive eye care	Urban or peri-urban outreach clinics	Schools, children’s programs	Remote communities; locations lacking specialist
Primary Strengths	Full clinical capacity; on-site diagnostics	Flexible, adaptable, cost-efficient compared to FEMU	Addresses pediatric disparities; integrates with schools	Extends subspecialty reach; efficient triage
Primary Limitations	Expensive; complex logistics; follow-up gaps; depend on academic partner and private funding most of the times	Limited diagnostics; follow-up gaps; depend on academic partner and private funding most of the times	Requires school coordination; depend on academic partners; follow-up gaps	Requires connectivity; may delay care if asynchronous; follow-up gaps

## Data Availability

No new data were created or analyzed in this study. Data sharing is not applicable to this article.

## Abbreviations

The following abbreviations are used in this manuscript:

MEU	Mobile Eye Units
FEMUS	Fully Equipped Mobile Units
SMOU	Semi-Mobile Outreach Units
SBVMU	School-Based Vision Mobile Units
HTOU	Hybrid Tele-Ophthalmology Units
OHSU	Oregon Health & Science University
EHR	Electronic Health Record
UPMC	University of Pittsburgh Medical Center
U	University
UCLA	University of California Los Angeles
IUSOC	Indiana University Student outreach Clinic Eye Program (IUSOC)
KEI	Kresge Eye Institute
eCSC	Effective Cataract Surgical Coverage
MESU	Mobile Eye Surgical Units

## Appendix A. U.S Residency Programs with Mobile Eye Units (MEUs)

**Table A1:** Provides a state-by-state list of all U.S. ophthalmology residency programs reviewed in this study, indicating whether each program operates or partners with a Mobile Eye Unit (MEU). For programs with an active MEU, relevant website links are provided when available. This appendix serves as a reference to complement Figure 2 and the Results section, where residency-affiliated MEU involvement is summarized. Programs listed as “Yes” demonstrate participation in mobile outreach initiatives such as school-based clinics, teleophthalmology vans, community screening programs, or fully equipped mobile clinics. **U: University, MEU: Mobile Eye Unit**.

State	Name of the Program	MEU?	Link
**Alabama**	U Alabama	No	
**Arkansas**	U Arkansas	Yes	https://news.uams.edu/2024/12/20/uams-govision-van-rolls-out-screens-first-patients/ (Accessed on 1 July 2025)
**Arizona**	U Arizona	No	
**California**	Stanford University	Yes	https://med.stanford.edu/ophthalmology/patient_care/tele-eyecare.html#remote_diabetic_eyecareprogram (Accessed on 1 July 2025)
	U California-Irvine	Yes	https://ophthalmology.uci.edu/community/eye-mobile-children (Accessed on 1 July 2025)
	U California-LA	Yes	https://www.uclahealth.org/departments/eye/mobile-eye (Accessed on 1 July 2025)
	U California-LA/EyeSTAR	Yes	https://www.uclahealth.org/departments/eye/mobile-eye (Accessed on 1 July 2025)
	U California—San Diego	Yes	https://shileyeye.ucsd.edu/about-us/shiley-eyemobile (Accessed on 1 July 2025)
	U California—SF	Yes	https://ophthalmology.ucsf.edu/log-on-the-doctor-will-see-you-now/eyevan/ (Accessed on 1 July 2025)
	USC Roski Eye Institute	No	
	U California—Davis	No	
	Loma Linda University	No	
	CPMC-San Francisco	No	
	U Colorado	No	
	Yale New Heaven Medical Center	No	
	Georgetown U/Wash Hosp	No	
	Howard U-Wash, DC	No	
**Colorado**	George Washington U	No	
**Connecticut**	U Miami/Bascom Palmer	Yes	https://med.miami.edu/about-us/community-outreach (Accessed on 1 July 2025)
**District of Columbia**	University of South Florida	No	
	U Florida-Gainesville	Yes	Not available (Accessed on 1 July 2025)
	University of Florida College of Medicine-Jacksonville	No	
**Florida**	Broward health	No	
	Emory University	No	
	Med C Georgia	No	
	Loyola U/Hines VA Hosp	No	
	Cook county-Chicago	No	
**Georgia**	University of Chicago	No	
	Rush Medical Center Northwestern University	No	
**Illinois**	Illinois Eye and Ear Infirmary	No	
	Indiana University	No	
	U Iowa	No	
	U Kansas	No	
	U Kentucky	Yes	https://ukhealthcare.uky.edu/kentucky-childrens-hospital/services/mobile-clinic (Accessed on 1 July 2025)
**Indiana**	U Louisville	No	
**Iowa**	LSU—Shreverport	No	
**Kansas**	LSU—New Orleans	No	
**Kentucky**	Tulane University	No	
	Sinai Hospital—Baltimore	No	
**Louisiana**	Wilmer-Johns Hopkins	No	
	U Maryland	No	
	Tufts/New England Eye Center	No	
**Maryland**	U Massachusetts Chan Medical School	No	
	Harvard Medical School—Mass, Eye & Ear	No	
	Boston University	No	
**Massachusetts**	Henry Ford Hospital-Detroit	No	
	U Michigan	No	
	Beamount Health-Royal Oak	No	
	Beamount Health-Taylor	No	
**Michigan**	Ascension Macomb-Oakland	No	
	Kresge Eye Institute-Wayne State	No	
	U Minnesota-Minneapolis	No	
	Mayo Clinic	No	
	University of Mississippi	No	
	Washington University	No	
**Minnesota**	St. Louis University	No	
	U Missouri—Columbia	No	
**Mississippi**	U Missouri—Kansas City	No	
**Missouri**	U Nebraska	No	
	Dartmouth-Hitchcock	No	
	Rutgers New Jersey Medical School	No	
	Columbia U/Harkness	Yes	https://www.cuimc.columbia.edu/news/tele-ophthalmology-van-pioneers-blindness-prevention-effort#:~:text=Through%20the%20department’s%20new%20tele,macular%20degeneration%2C%20and%20diabetic%20retinopathy. (Accessed on 1 July 2025)
**Nebraska**	NY Presb. Hosp-Weill MC/Cornell U	Yes	https://eye.weillcornell.org/education-training/residency-program/international-and-community-service (Accessed on 1 July 2025)
**New Hampshire**	University of Rochester Flaum Eye Institute	Yes	https://www.urmc.rochester.edu/eye-institute/healthy-eyes/mobileyes (Accessed on 1 July 2025)
**New Jersey**	BronxCare Health System	Yes	https://www.bronxcare.org/our-services/ophthalmology/ophthalmology-residency-program/community-service (Accessed on 1 July 2025)
**New York**	SUNY—Downstate	Yes	Not available (Accessed on 1 July 2025)
	Albany Medical College	No	
	SUNY—Upstate (Syracuse)	No	
	SUNY—Buffalo	No	
	New York Eye & Ear Infirmary	No	
	Albert Einstein College of Medicine	No	
	Northwell/Hofstra—No—No website	No	
	Nassau University MC	No	
	New York Medical College	No	
	St. John’s Episcopal Hospital	No	
	SUNY—Stony Brook	No	
	NYU Grossman	No	
	NYMC at Jamaica Hospital	No	
	Duke University	Yes	https://dukeeyecenter.duke.edu/news/remote-diagnosis-becomes-reality-new-mobile-imaging-van (Accessed on 1 July 2025)
	U North Carolina	Yes	https://shorturl.at/eEC7s (Accessed on 1 July 2025)
	Wake Forest University	No	
	Cleveland Clinic-Cole Eye Institute	Yes	https://my.clevelandclinic.org/departments/eye/services/pediatric-ophthalmology#community-outreach-tab (Accessed on 1 July 2025)
**North Carolina**	Ohio State university	Yes	https://medicine.osu.edu/departments/ophthalmology/community-and-global-outreach/teleophthalmology (Accessed on 1 July 2025)
	Case Western Reserved University	No	
	Kettering Health Network	No	
**Ohio**	Cincinnati University	No	
	U Oklahoma	No	
	Oregon HSU (Casey Eye Institute Outreach)	Yes	https://www.ohsu.edu/casey-eye-institute/casey-community-outreach-program-0 (Accessed on 1 July 2025)
	U Pittsburgh	Yes	https://eyeandear.org/eyes-on-wheels/ (Accessed on 1 July 2025)
	Scheie Eye Institute/U Penn	Yes	https://www3.pennmedicine.org/departments-and-centers/ophthalmology/about-us/news/department-news/scheie-offers-free-diabetic-eye-screenings-to-community (Accessed on 1 July 2025)
**Oklahoma**	Nazareth Hospital Ophthalmology Residency	No	
**Oregon**	Temple U—Philadephia	No	
**Pennsylvania**	Wills Eye Hospital	No	
	Penn State U—Hershey	No	
	Geisinger MC	No	
	Philadelphia College of Osteopathic Medicine	No	
	U Puerto Rico	No	
	Brown University	No	
	Med U South Carolina	No	
	U South Carolina	No	
**Puerto Rico**	Vanderbilt University Medical Center	No	
**Rhode Island**	U Tennessee-Memphis	No	
**South Carolina**	U Texas-San Antonio	Yes	https://lsom.uthscsa.edu/ophthalmology/education/medical-student-education/opthalmology-interest-group/ (Accessed on 1 July 2025)
	U Texas/Methodist—Galveston	No	
**Tennessee**	UT Southwestern-Dallas	No	
	U Texas-Houston	No	
**Texas**	UT Austin Dell Medical School	No	
	Texas A&M/Baylor Scott & White	No	
	Baylor College of Medicine/Cullen Eye Institute	No	
	Texas Tech University	No	
	U Utah	Yes	https://healthcare.utah.edu/moran/outreach/community-clinics/ https://www.youtube.com/watch?v=-MeUEqdgKcI (Accessed on 1 July 2025)
	Eastern Virginia MS	No	
	U Virginia	No	
	Virginia Commonwealth University	No	
**Utah**	U Washington	No	
**Virginia**	West Virginia University Eye Institute	Yes	https://medicine.hsc.wvu.edu/eye/outreach/appalachian-vision-outreach-program-avop (Accessed on 1 July 2025)
	Med C Wisconsin	No	
	U Wisconsin	No	
**Washington**			
**West Virginia**			
**Wisconsin**			

## Appendix B. Case Examples Illustrating Operational Characteristics of Mobile Eye Units (MEUs)

**Table A2:** Summarizes representative case examples for each Mobile Eye Unit (MEU) model identified in this review. The table compares programs across key operational domains, including population served, services provided, equipment used, follow-up mechanisms, and primary implementation challenges. These examples illustrate the diversity of MEU structures in North America and highlight the practical considerations that shape program design and performance. FEMU: Full-Equipped Mobile Units, SMOU: Semi-Mobile Outreach Units, SBVMC: School-Based Vision Mobile Units, HTOU: Hybrid Tele-Ophthalmology Units, OHSU: Oregon Health & Science University, UPMC: University of Pittsburgh Medical Center, DRS: Diabetic Retinopathy Screening Program.

Program Name	Affiliation	Model Type	Population Served	Services Provided	Equipment Used	Follow-Up Mechanism	Key Challenges
**Casey Eye Institute Outreach Bus**	**OHSU**	FEMU	Underserved communities in Oregon and Pacific Northwest	Full eye exams, diabetic eye care, glaucoma screening, refractions, retinal imaging, referrals	Slit lamps, fundus cameras, autorefractors, indirect ophthalmoscopes, EHR access	On-site triage, referrals to OHSU or partners	Episodic care, sustainability, follow-up
**UPMC eyeVan**	**UPMC**	FEMU/SMOU	Low-income, underserved communities in Western PA	Eye-exams, retinal imaging, glasses, refraction, OCT, pressure checks	Phoropter, slit lamp, indirect ophthalmoscope, fundus camera, OCT, mobile internet	Patient navigators, scheduled follow-up at UPMC clinics	Sustainability, logistics, equipment maintenance
**The Health Wagon**	**Independent/partnerships**	FEMU/SMOU	Low-income, rural Appalachian populations	Primary care + vision screening, diabetic eye screening, cataract evals	Portable ophthalmic equipment	Occasional referrals, volunteer specialists	Episodic, continuity, volunteer-dependence
**UCLA Mobile Eye Clinic (UMEC)**	**UCLA Stein Eye Institute**	SMOU	Homeless, uninsured, low-income children and adults	Basic exams, refraction, glasses	Portable visual acuity charts, autorefractors	Referral for advanced care	Limited diagnostics, dependent on partnerships
**Eyes on Wheels**	**UPMC Vision Institute**	SMOU	Uninsured and underinsured in Western PA	Full eye exams, referrals, screenings	Portable slit lamps, autorefractors, VA charts, tonometers	UPMC referral pathways, case navigation	Volunteer reliance, patient follow-through
**IUSOC Eye Program**	**Indiana U School of Medicine**	SMOU	Uninsured urban residents	Eye exams, pressure checks, referrals	Slit lamps, autorefractors, VA charts	Referral to partner clinics	Limited clinic frequency, volunteer dependence
**Vision Detroit Project**	**Kresge Eye Institute**	SMOU	Underserved in Detroit (African American, immigrant)	Full eye exams, diabetic & glaucoma screening, glasses	Portable diagnostic equipment	Partner navigation, referrals for surgery	Funding, mobile logistics, patient mobility
**Vision First**	**Cleveland Clinic Cole Eye Institute**	SBVMC	Public school students in Cleveland	Vision screening, cycloplegic refraction, glasses, referrals	School-based van, slit lamps, indirect ophthalmoscopes	Coordinator + referral to peds ophth	Continuity, follow-up, parental consent
**Conexus Mobile Vision Clinic**	**Conexus (nonprofit)**	SBVMC	Students in underserved areas of Virginia	Full eye exams, glasses	Mobile clinic with comprehensive setup	School delivery + parent coordination	Scheduling, space, longitudinal care
**Ypsilanti School-Based Eye Clinic**	**U Michigan Kellogg Eye Center**	SBVMC	Low-income, minority students in Ypsilanti	Eye exams, pressure check, glasses	School-based equipment	School nurses + referrals	School calendar, sustainability
**Columbia Teleophthalmology Unit**	**Columbia University Irving Medical Center**	HTOU	Public housing residents, uninsured adults in NYC	Fundus photography, asynchronous image review, navigation	Non-mydriatic cameras, VA charts	Navigator follow-up + scheduling	Connectivity, image quality, coordination
**British Columbia DR Teleunits**	**BC Provincial Health Services + Indigenous orgs**	HTOU	Remote Indigenous communities	DR screening, image upload + asynchronous review	Fundus cameras, upload tech	Local provider reports + referrals	Infrastructure, cultural barriers
**DRSP-UPMC**	**UPMC Vision Institute**	HTOU	UPMC affiliated facilities and Indigen clinics	DR screening, image upload + asynchronous review	Fundus cameras, upload tech	Local provider reports + referrals	Infrastructure, no live interactions, system capacity
